# Hospital Needs and Hospital Appeals

**Published:** 1922-12

**Authors:** 


### HOSPITAL NEEDS AND HOSPITAL APPEALS.

### Hospitals with 150 Beds and Upwards.

**BROMPTON HOSPITAL FOR CONSUMPTION AND DISEASES OF THE CHEST.**—With its sanatoria and convalescent home at Chobham Ridges, this hospital has nearly 500 beds, all of which are constantly occupied. During 1921, 2,949 in-patients and 36,575 out-patients received treatment at this institution. The annual expenditure is over £80,000, of which only £5,000 is assured. Donors of £52 10s., and annual subscribers of £5 5s. become Governors, and may recommend one in-patient and eight out-patients every year. The secretary, Mr. Frederick Wood, is making a special appeal for new annual subscribers, and sincerely hopes that the public will respond generously. Contributions may be sent to him at the Hospital for Consumption, Fulham Road, S.W.3.

**CHARING CROSS HOSPITAL.**—This institution is situated in the very heart of the metropolis, and in the course of the year treats nearly one hundred thousand casualties and out-patients. The hospital has also some 250 beds for in-patients. The motto of the Council is "Efficiency with Economy," and owing to its enforcement, the institution to-day is free of debt and in full working order. Not a single bed has been closed, nor one section of the work curtailed. It is hoped that this satisfactory state of affairs may continue, but increased support is absolutely necessary, as during the coming year a new maternity department, a new children's ward, and a new nurses' home are urgently required. Any gift, however small, will be gratefully acknowledged by the Secretary-Superintendent, Charing Cross Hospital, W.C.2.

**CITY OF LONDON HOSPITAL FOR CHEST DISEASES.**—The annual expenditure of this hospital is £29,000, while its assured income amounts to only £1,000. Consequently, the difference of £28,000 must be met by voluntary support, and a very strong appeal is made for new annual subscriptions and donations. The sum of £1,000 will endow a bed and £500 a cot, and Mr. George Watts, the secretary, will be thankful for any assistance which may be addressed to him at the hospital, Victoria Park, E.2.

**GUY'S HOSPITAL.**—Founded in 1724, by Thomas Guy, this great institution has to-day no less than 614 beds, and annually treats 9,261 in-patients, and 116,801 out-patients, while out-patient attendances number 510,528. The annual cost of maintenance is £169,028, of which £100,000 must be obtained by means of voluntary subscriptions and donations, and help is earnestly solicited by the treasurer, Viscount Goschen, and should be addressed to him at Guy's Hospital, London Bridge, S.E.1.

**HOSPITAL FOR SICK CHILDREN.**—This hospital will close the year's work with a debt to the bankers of £10,000, and so far as can be estimated, there will be a deficit of a similar amount between income and necessary expenditure for 1923. These deficits are after making due allowance for the sums received and anticipated from the Voluntary Hospitals Commission and the distribution of the proceeds of the Voluntary Hospitals Combined Appeal. Further annual subscriptions and donations will be thankfully received by Mr. James McKay, at the hospital, Great Ormond Street, London, W.C.1.

**KING'S COLLEGE HOSPITAL.**—During 1922 the ordinary income of the hospital has shown a very considerable decrease, due primarily to the diversion of support to the Combined Appeal. Loans have been raised upon the resources of the hospital to the utmost limit, and it is anticipated that, in addition to the present debt of £80,000, a further £20,000 will be incurred by the end of the year. To assist the funds of the institution the experiment of using the empty wards for private patients has proved useful. Patients have paid a sum at least covering the cost of maintenance, and in some cases there has been a small margin available to bear the overhead charges upon the empty wards, but this is by no means sufficient to meet the needs of this great institution, and further assistance is urgently required, and will be gratefully received by the secretary of the hospital, Denmark Hill, Camberwell, S.E.5.

**LONDON FEVER HOSPITAL.**—Established in 1802, this hospital receives no help whatever from the rates. The income is derived from voluntary sources, supplemented by patients' payments, which in the case of ward patients cover only a small proportion of the actual cost. During the year 1921, 1,088 patients were treated, all suffering from infectious diseases. Further help is needed to assist this, one of London's oldest charities, to carry on the work, of which the annual cost is about £25,000, and at least £10,000 of this sum must be collected in the New Year. Subscriptions and donations should be sent to Commander T. J. Farrell, D.S.C., secretary, London Fever Hospital, Islington, N.1.

**LONDON HOMŒOPATHIC HOSPITAL.**—This hospital is much handicapped in the treatment of some 1,600 in-patients and 12,000 out-patients annually by a totally inadequate subscription list. Although the past year has shown a slight financial improvement, it is by no means commensurate with the demands made upon the institution by an ever-increasing number of patients. A strong appeal is made for help to replace the deficiency arising by reason of the death of many subscribers and the enforced withdrawal of others. Financial help should be forwarded to Mr. Edward A. Attwood, secretary, London Homœopathic Hospital, Great Ormond Street, Bloomsbury, W.C.1.

**LONDON HOSPITAL.**—The immense work which this well-known hospital accomplishes in the course of the year is of the utmost service to the community, and merits the generous support of the charitable public. The cost of upkeep is over £256,992, most of which is obtained from voluntary subscriptions; consequently it is hoped that this institution will receive a larger measure of support in the coming year, in order that it may continue and, if possible, enlarge its good work. Subscriptions and donations may be sent to Mr. E. W. Morris, the house governor, at the Hospital, Whitechapel Road, E.1.

**LONDON LOCK HOSPITAL.**—By December 31, 1922, this institution requires the sum of £3,618 to pay for the new out-patients' department and mortuary now rapidly approaching completion. The plans were passed by King Edward's Hospital Fund, and the whole scheme has had its cordial approval. Over 3,000 women and children attend as out-patients each year at Harrow Road. A donation of £500 to the Building Fund constitutes Life Governorship. Address the secretary, 283 Harrow Road, W.9.

**METROPOLITAN HOSPITAL.**—Situated in one of the poorest districts of London, this hospital is urgently in need of financial help. In addition to this, funds are wanted for a new nurses' home, most urgently required for the proper accommodation of the staff, and special donations are solicited for the carrying out of this important and necessary work. Subscriptions and contributions should be addressed to Mr. Herbert F. Rutherford, secretary, Metropolitan Hospital, Kingsland Road, E.8.

**NATIONAL HOSPITAL FOR THE PARALYSED AND EPILEPTIC.**—This is the oldest and largest hospital in the country for the treatment of diseases of the nervous system. Patients are received from all parts of the British Isles and from distant places abroad. Last year, by special effort, the board of management obtained just enough money to pay expenses, which are approximately £40,000 a year. This year it is hoped that the grants from the Combined Appeal may bring about the same result, but the constant efforts to obtain funds for maintenance do not provide for improvements to the hospital. An out-patient theatre is badly needed; the in-patient theatre also requires alteration, and for the whole scheme a sum of about £5,000 is needed. Mr. Godfrey H. Hamilton will welcome any assistance, addressed to him at the Hospital, Queen Square, Bloomsbury, W.C.1.

**NORTHAMPTON GENERAL HOSPITAL.**—A much-needed central heating station has been installed recently at this institution at a cost of £18,500, of which the sum of £5,150 is still owing. Other alterations also are necessary, the most urgent being a new isolation block, which it is estimated will cost £6,000. Donations are earnestly solicited towards these objects, and will be gratefully received by Mr. C. S. Risbee, secretary-superintendent, Northampton General Hospital, Northampton.

**ROYAL FREE HOSPITAL.**—An urgent appeal is made for the sum of £200,000 to extend the existing buildings on an adjoining site recently presented to the hospital. The intention of the authorities being to provide the much-needed additional accommodation for the treatment of patients, as well as for undergraduate and post-graduate medical education. Grants and the allocation of funds to these objects are specially solicited, and may be sent to Mr. Reginald R. Garrett, secretary, Royal Free Hospital, Gray's Inn Road, W.C.1.

**ROYAL NORTHERN GROUP OF HOSPITALS.**—This group of hospitals comprises the Royal Northern Hospital, Holloway; the Royal Chest Hospital, City Road; a Hospital of Recovery at Southgate; and a convalescent home at Clacton-on-Sea. The whole organisation has 375 beds, and at the main building at Holloway there are private, contributory, and general wards, and 17 departments for the treatment of special diseases. For the past few years the task of carrying on the work of this group has been exceedingly difficult owing to lack of adequate financial support. Those officials responsible for the management have made strenuous efforts to keep open all wards to meet the needs of the million people, in the 70 square miles of North London, who look to these institutions in time of accident or serious illness. There is now a deficit of £36,000, and the lowest possible estimate for efficient working during 1923 is £85,000. Help should be sent to Mr. Gilbert G. Panter, at the Royal Northern Hospital, Holloway Road, N.7.

**ST. BARTHOLOMEW'S HOSPITAL.**—To continue the work of London's oldest hospital the sum of £100 a day is required each day through the year, and there is an average annual deficit of £40,900 of the £153,357 necessary to maintain to the fullest capacity the 686 beds of this great institution. The treasurer will be glad to give information as to the sum necessary for the naming of wards or beds as memorials for perpetuity. Contributions will be gratefully received by the Right Hon. Lord Stanmore, St. Bartholomew's Hospital, E.C.1.

**ST. GEORGE'S HOSPITAL.**—Over 30,000 patients are treated annually at this hospital, in addition to the 850 received at the convalescent branch at Wimbledon, and the committee most urgently appeal for Christmas donations to help towards the reduction of the deficit on the current year's work and a debt to bankers of £16,600. Contributions will be thankfully received by Mr. James M. Churchfield, secretary-superintendent, St. George's Hospital, Hyde Park Corner, S.W.1.

**ST. MARY'S HOSPITAL.**—The regular sources of income yield about £20,000, and to carry on the work of this hospital efficiently on its present basis throughout the year costs about £70,000. Therefore, to maintain the hospital to its fullest capacity a further sum of £50,000 is required, and a strong appeal is made for contributions towards the liquidation of this formidable deficit. Donations and subscriptions may be sent to the treasurer, or the secretary, Mr. W. Parkes, St. Mary's Hospital, Praed Street, Paddington, W.2.

**SEAMEN'S HOSPITAL SOCIETY (Dreadnought).**—The old "Dreadnought," by which name the Seamen's Hospital Society is known to sailors all the world over, continues its good work of caring for sick and injured mariners. Ordinary ailments are treated in the Dreadnought Hospital, Greenwich (250 beds), and at the Albert Dock Hospital (50 beds), while the Society cares for those who are suffering from the insidious diseases contracted in the Tropics at their Hospital for Tropical Diseases, to which is attached the London School of Tropical Medicine. There is also the Angas Convalescent Home at Cudham, with 30 beds, where patients recovering from serious illness may go to rest awhile before returning to their arduous calling. In all over 21,000 patients were treated in the five establishments of the Seamen's Hospital Society during the year 1921. The expenditure is upwards of £60,000 per annum, and the Board make an earnest appeal for these men, to whom the country is so much indebted. Secretary, Sir James Michelli, C.M.G., Seamen's Hospital Society, Greenwich, S.E.10.

**SOUTH DEVON AND EAST CORNWALL HOSPITAL.**—Funds are urgently needed to free this charity from an estimated deficit of £5,000 on the current year's work; to re-open two wards containing 44 beds, which have been closed since the 1st of January; the development of laboratories for bio-chemical investigation; X-ray and electro-therapeutic departments; and the introduction of steam cooking and the renovation of the kitchen. Donations should be sent to Mr. P. J. Langdon, secretary, South Devon and East Cornwall Hospital, Plymouth.

**WESTMINSTER HOSPITAL.**—During the year, 2,618 in-patients and 16,195 out-patients were treated at this institution. To carry on the work on its present basis £45,000 is required annually, of which there is an assured income of only £23,000, leaving a balance of £22,000 to be obtained from voluntary contributions. In addition, £15,000 is required to meet much needed repairs and for the provision of a new operating theatre. It is hoped, therefore, that the public will realise the importance of coming to the assistance of this hospital by giving immediate support in the form of donations and subscriptions, which should be forwarded to Mr. Charles M Power, secretary, Westminster Hospital, Broad Sanctuary, S.W.1.

### Hospitals with 50 Beds and under 150.

**CANCER HOSPITAL (FREE).**—This special hospital, which is devoted to one of the most prevalent and dreaded of human diseases, is urgently in need of funds for general purposes, and also to carry on its important work of research into the causes and alleviation of cancer. It therefore has distinct claims upon the consideration of the benevolent public, and it is hoped that generous help may be forthcoming to support this most worthy of institutions. Contributions should be sent to the secretary, Mr. James Courtney Buchanan, The Cancer Hospital, Fulham Road, Brompton, S.W.3.

**CHELSEA HOSPITAL FOR WOMEN.**—Special efforts are being made by this hospital to liquidate a debt of £2,500, and also to increase the regular income to meet normal expenditure. A further sum of £12,000 is required to complete the fund for the building of the nurses' home, which is so essential since it will release for the use of patients some 50 beds in the hospital which are now occupied by the staff. Contributions towards these objects will be gratefully received by the honorary treasurer or secretary, Mr. Herbert A. Jennings, the Chelsea Hospital for Women, Arthur Street, Chelsea, S.W.3.

**CITY OF LONDON MATERNITY HOSPITAL.**—Established in Aldersgate Street, in the East-end of London, in 1750, this is the oldest maternity hospital in the kingdom, and assists the poorest married women and single women with their first child. The sum of about £10,000 is required towards extensive enlargements which have become imperative in various departments. This expense will necessitate the incurrence of a further debt to bankers unless the sum can be raised by immediate donations and the committee of management will be glad to receive contributions, however small, to avoid this further liability. Address the secretary, City of London Maternity Hospital, 102, City Road, E.C.1.

**CLAPHAM MATERNITY HOSPITAL.**—The institution was not in debt on December 31, 1921, but of late years the amount subscribed by private persons has been small, and more guinea subscribers are desired in order to balance receipts and expenditure. The hospital started in a rented house, but now stands on its own half-acre of freehold ground. A new building, suitable for the reception of 50 mothers and babies, has been erected recently, and a small addition for nurseries and ward-kitchens would add to the value of the 15 new wards. The committee is making a special effort to collect £1,000 for this purpose, and the help of everyone interested in mothers and babies is solicited by Miss Marion Ritchie, secretary, Clapham Maternity Hospital, Jeffreys Road, Clapham, S.W.4

**EVERSFIELD HOSPITAL FOR CONSUMPTION AND DISEASES OF THE CHEST.**—This institution is greatly in need of help, over £22,000 being required for extensions and endowments and the reduction of mortgages. Donations may be sent to Mrs. E. S. Gambier, B.A., Eversfield Hospital, St. Leonards-on-Sea.

**HAMPSTEAD GENERAL AND NORTH-WEST LONDON HOSPITAL.**—This hospital is in debt to the extent of £9,000, in addition to a deficit of £3,500 between income and expenditure on the current year's work. Unless immediate help is forthcoming, the hospital will close the year with a debt of £12,500, and an urgent appeal is made to enable the committee to clear this sum so that the hospital may commence the New Year untrammelled by this responsibility. Contributions may be sent to the secretary, Mr. Harold Wigg, Hampstead General and North-West London Hospital, Haverstock Hill, N.W.3.

**MILDMAY MISSION HOSPITAL.**—Situated in the East-end of London, the Mildmay Mission Hospital has 52 beds, and last year treated 750 in-patients, in addition to 36,000 out-patients; the expenditure involved in the year's working was £8,700. To carry on the activities of the hospital free of debt during the coming year a sum of £11,000 will be required. Funds are needed also for the installation of central heating, electric light, and additional accommodation for the nursing staff, and a very strong appeal is made for donations and subscriptions towards attaining these most necessary objects, while the committee will welcome new subscribers to assist towards the increased expenditure necessary to carry on the work of the hospital. All donations should be sent to the secretary at the hospital, Austin Street, Bethnal Green, E.2.

**MILLER GENERAL HOSPITAL FOR SOUTH-EAST LONDON.**—Largely owing to the help which has been already received from the Combined Appeal for the Hospitals of London, it is hoped that expenditure will be met this year, but a sum of £27,000 is required annually, and as assured income is only a little over £1,000, each year commences with considerable anxiety. There is much need for the extension of this hospital so that the shortage of hospital beds in the S.E. district of London may be met, and King Edward's Hospital Fund has approved an extension up to 336 beds, costing about £50,000. Subscriptions and donations will be gladly received by Mr. Harry A. Bone, at the hospital, Greenwich Road, S.E.10.

**MOUNT VERNON HOSPITAL FOR TUBERCULOSIS.**—At the out-patients' department, Fitzroy Square, W., this hospital treats all deserving persons suffering from tuberculosis and diseases of the lungs and heart, and the secretary, Mr. J. W. Morton, earnestly appeals for subscriptions and donations to carry on the good work which this institution is doing on behalf of the suffering poor. All help of a financial character should be addressed to him at the Mount Vernon Hospital, 7, Fitzroy Square, W.1.

**POPLAR HOSPITAL FOR ACCIDENTS.**—This hospital is making an effort to raise £20,000, which is required for general expenses, and a further £12,000 to complete the estimated total of £50,000 required for rebuilding purposes, while an additional £20,000 is necessary for the formation of an endowment fund. The committee earnestly trust that these deserving objects will appeal to the public, and that a ready response will be made in the form of donations and subscriptions, which will be gladly received by the secretary, Mr. D. H. Lindsay, Poplar Hospital for Accidents, East India Dock Road, Poplar, E.14.

**PRINCE OF WALES'S GENERAL HOSPITAL.**—Tottenham is one of the poorest districts of Outer London, and this institution requires annually £23,000 to maintain its 125 beds. Over 30,000 patients are treated each year, and are received without subscribers' letters, the only passport to secure the services of the hospital being necessity. There is a debt at the bankers of £10,000, and an urgent appeal is made for donations towards the liquidation of this sum, which it is hoped will be forthcoming before the end of the year. Mr. F. W. Drewett, F.C.I.S., Director, Prince of Wales's General Hospital, Tottenham, N.15.

**QUEEN CHARLOTTE'S LYING-IN HOSPITAL.**—Nearly 2,000 women are received into the wards of this institution every year, and over 2,000 are attended by the hospital midwives and nurses in their own homes. In addition, some 5,000 patients attend the ante-natal and child welfare department. In the midwifery training school about 150 students are trained in midwifery annually, and nearly 200 women are trained as midwives and maternity nurses. Great efforts have been made to free the hospital of debt, but upwards of £15,000 is still required. Moreover, the much needed enlargement of the hospital, which was about to be commenced on the outbreak of war, has had to be postponed until more prosperous times. Donations will be gratefully received by the secretary, Mr. Arthur Watts, at the hospital, Marylebone Road, N.W.1.

**QUEEN'S HOSPITAL FOR CHILDREN.**—To continue its good work among the children of Hackney, and to maintain adequately its 164 beds and convalescent home at Bexhill, this hospital is greatly in need of increased support in the form of new annual subscriptions, which will be gladly welcomed by the secretary, The Queen's Hospital for Children, Hackney Road, E.2.

**QUEEN MARY'S HOSPITAL FOR THE EAST END.**—This hospital is appealing for donations towards the liquidation of an outstanding liability of £10,000. It is also in need of increased support to enable it to carry on adequately its work in one of the largest boroughs in England, with a population exceeding a million souls. Contributions should be sent to Mr. A. W. Scrivener, secretary, Queen Mary's Hospital, West Ham Lane, West Ham, E.15.

**ROYAL WATERLOO HOSPITAL FOR CHILDREN AND WOMEN.**—This institution owes its bankers £9,500, and of this sum, under an order of the Charity Commissioners, £2,850 must be repaid before May, 1923. The estimated expenditure for the New Year is £15,000, of which about £12,000 only is assured, leaving a balance of £3,000 to be raised from voluntary sources. The committee consequently appeal for £5,850 to meet immediate needs, and contributions will be gladly welcomed by the secretary, Royal Waterloo Hospital for Children and Women, Waterloo Road, S.E.1.

**ST. ANDREW'S HOSPITAL.**—An urgent appeal is made for increased funds for maintenance, and also for the reduction of the building debt and the liquidation of a deficit of over £1,462, which is crippling the work of this institution. Donations should be sent to the secretary, St. Andrew's Hospital, Dollis Hill, London, N.W.2.

**ST. JOHN'S HOSPITAL, LEWISHAM.**—In view of the extra cost of maintenance which will be incurred with the opening of the new wards, the board earnestly appeal for annual subscriptions. Special donations also are needed towards the cost of the children's and women's wards, especially as the King's Fund is unable to afford the hospital any financial support. Mr. Francis M. Fryatt, hon. secretary, St. John's Hospital, Morden Hill, S.E.13.

**ST. MARK'S HOSPITAL FOR CANCER.**—The committee of management earnestly appeal for contributions to meet the cost of the new X-ray equipment recently installed. For many years the absence of a thoroughly up-to-date X-ray department has restricted the surgical staff in their efforts to localise and treat cases of cancer in their early stages, and it was felt that the installation of modern apparatus could be no longer delayed. An earnest appeal is made for assistance towards meeting the expenditure of £530, and contributions will be gratefully received by the secretary, St. Mark's Hospital, City Road, E.C.1.

**ST. PETER'S HOSPITAL.**—The necessary structural alterations of this hospital, which have cost the sum of £2,246, are now completed, and the committee earnestly appeal for help to pay off the money borrowed from the bankers for this undertaking. Contributions should be sent to Mr. Irwin H. Beattie, secretary, St. Peter's Hospital, Henrietta Street, Covent Garden, London, W.C.2.

**SAMARITAN FREE HOSPITAL FOR WOMEN.**—An appeal is made for help to enable the committee to proceed with the necessary external repairs and the re-wiring of the whole of the electric light installation. This will cost £2,200, but financial difficulties have prevented the authorities so far from putting the work in hand. So serious are the defects in the external structure that to delay longer is a matter of grave concern, and any contribution, however small, to enable the work to be commenced without further delay, will be most thankfully received by the secretary, Samaritan Free Hospital for Women, Marylebone Road, N.W.1.

**SOUTH-EASTERN HOSPITAL FOR CHILDREN.**—To maintain efficiently its 60 cots, which are constantly occupied, this hospital commenced the year with a debt to bankers of £1,100, and an appeal is made for donations to free the institution of this liability. Contributions also are needed towards the rebuilding of the out-patients' department, which is inadequate to accommodate the 25,000 out-patients treated annually. Help towards these objects should be sent to Mr. W. Mason, hon. secretary, 83, Dacres Road, Forest Hill, S.E.23.

**SOUTH LONDON HOSPITAL FOR WOMEN.**—The work of extending the in-patient department by altering and adding to the adjoining building presented for the purpose is now in progress. This extension will provide 35 additional beds for surgical cases, and accommodation for nursing and domestic staffs. The total cost of the alterations will amount to £15,000, of which £12,000 is in hand, leaving a balance of £3,000 to be obtained. An appeal is made for donations towards this outlay, and also for new annual subscriptions and donations to meet the increased annual expenditure on upkeep. Contributions should be sent to the secretary, South London Hospital for Women, South Side, Clapham Common, S.W.4.

**VICTORIA HOSPITAL, CHELSEA.**—During the year 1921 this institution proved to be one of the most economically managed children's hospitals in London. Nevertheless, it finished the year with a deficit of £7,529, and is now making strenuous efforts to clear itself of debt by the end of the year. Additional accommodation and provision for physio-therapy treatment have been urgently needed, and these requirements are being met in the new "Princess Mary Home and Physio-Therapy Department," adjoining the hospital. The institution now has 132 beds in London, and 50 beds at the Victoria Convalescent Home, Broadstairs. Help in the form of donations and new annual subscriptions should be sent to Mr. H. G. Evered, secretary, Victoria Hospital for Children, Tite Street, Chelsea, S.W.3.

### Hospitals under 50 Beds.

**BELGRAVE HOSPITAL FOR CHILDREN.**—There is an overdraft on the bank of £3,000, and the committee appeal most earnestly for contributions to pay this debt off before the end of the year. Donations also are solicited towards the building of a new south wing, which will provide an extra 28 beds, badly needed owing to the largely increased attendance in the out-patients' department. During the year an additional ward of ten beds has been opened, and set apart specially for children requiring operations for tonsils and adenoids. Secretary, Mr. Thomas Clapham, Belgrave Hospital for Children, Clapham Road, S.W.9.

**CENTRAL LONDON THROAT, NOSE, AND EAR HOSPITAL.**—Every year this hospital has to raise the sum of £10,000 for upkeep alone. A pressing need is an up-to-date X-ray apparatus, and for this purpose it will be necessary to add an annexe to the existing lecture room at a cost of £1,000. In view of the fact that the out-patients number over 12,000 a year, and the in-patients close upon 1,000, it will be seen that such an equipment is essential to efficient diagnosis and treatment. Contributions should be sent to the secretary, at the Hospital, Gray's Inn Road, W.C.1.

**MILDMAY MEMORIAL HOSPITAL.**—At the end of October this hospital was in debt to the extent of £430, largely owing to the cost of the erection of fire staircases and balconies. The entire cost of these additions has to be met by the end of the year, and will amount to £1,220. Donations to meet this extraordinary outlay are earnestly solicited, and should be forwarded to the secretary, Mildmay Memorial Hospital, Newington Green Road, N.1.

**NATIONAL HOSPITAL FOR DISEASES OF THE HEART.**—This hospital is specially concerned in the study and treatment of diseases of the heart. It has 46 beds, and courses with lectures and clinical demonstrations are held for post-graduates. Instruction in electro-cardiography also is given. The sum of £8,000 is urgently required for extension of the out-patient department, and funds are much needed for research work. All contributions should be addressed to the secretary at the Hospital, Westmorland Street, W.1.

**PADDINGTON GREEN CHILDREN'S HOSPITAL.**—The hospital has 46 beds, and a Convalescent Home at Slough with 24 beds. The income for 1921 from all sources was £9,905, and the expenditure £11,342. Some 883 in-patients received treatment during the year, in addition to 11,342 new out-patients, while the total out-patients' attendances numbered about 40,000. The committee of management urgently appeal for new subscriptions, donations and legacies, to enable them to discharge the very heavy debt of £12,000 owing to bankers. Contributions will be thankfully received by the chairman, F. J. Walker, Esq., or the secretary, Mr. James A. Hamlin, at the Paddington Green Children's Hospital, London, W.2.

**ROYAL EYE HOSPITAL.**—This hospital has 22,000 new out-patients, and more than 850 in-patients are treated annually. The excess of expenditure over income is upwards of £1,000. New annual subscriptions and donations are therefore urgently needed. All contributions should be sent to the secretary, Royal Eye Hospital, St. George's Circus, Southwark, S.E.1.

**ROYAL WESTMINSTER OPHTHALMIC HOSPITAL.**—Founded 106 years ago, this hospital is greatly in need of funds for general maintenance purposes. This year, no less than 13,000 new patients have received attention, representing upwards of 30,000 attendances. Future needs are more beds and an annexe outside the London area, to which medical cases needing a long course of treatment may be sent. All subscriptions should be addressed to the secretary, Royal Westminster Ophthalmic Hospital, King William Street, West Strand, W.C.2.

**ST. JOHN'S HOSPITAL FOR DISEASES OF THE SKIN.**—Founded in 1863, this hospital is the largest of its kind in the kingdom, dealing as it does with 1,000 cases a week. Like other institutions, it is suffering from the present high prices of all commodities, and the board of management of this thoroughly deserving and necessary charity appeal earnestly for help. Cheques will be gratefully received by the president and treasurer, the Earl of Chesterfield, K.G., 49 Leicester Square, W.C.2, or by the secretary, at the same address.

### General Charities.

**DR. BARNARDO'S HOMES**—.The Charter of the Homes is "No Destitute Child Ever Refused Admission": the one and only qualification, therefore, for admission is destitution. In 1921, 13,540 boys and girls were dealt with; 1,349 being permanently admitted, and there are to-day 7,319 children under the care of the Homes. Some 27,740 boys and girls have been emigrated within the Empire, of which 98 per cent. are doing well. The institution comprises 156 separate branches and households, and the work formerly carried on at Her Majesty's Hospital. Stepney Causeway, is now transferred to the Australasian Hospital at the Girls' Village Home, Barkingside, Essex. where there are 1,400 girls always in residence; and to the John Capel Hanbury Hospital at the Boys' Garden City, Woodford Bridge, Essex, where are housed over 700 boys. Funds are urgently needed to support the work of this national charity, since, owing to the abnormal economic conditions prevailing since the war and the increase of unemployment, the demands upon Barnardos' resources are greater than ever. An earnest appeal is made for help, and will be welcomed at the Head Offices and Chief Ever-Open Door at 18-25 Stepney Causeway, E.1.

**IMPERIAL CANCER RESEARCH FUND.**—This society is carrying on a very important work in the interest of suffering humanity, and it is hoped that its investigations into the causes of cancer, and its efforts to find a cure for this dreaded disease, may not be hampered for want of adequate funds. Donations and subscriptions should be sent to the honorary treasurer, Examination Hall, 8-11 Queen Square, Bloomsbury, W.C.1.

**MENTAL AFTER-CARE ASSOCIATION.**—This charity, which was started in 1879, is the only one of its kind in the United Kingdom, and is sorely in need of increased financial support. There is an overdraft at the bank of nearly £500, owing to the large number of cases dealt with and the great difficulty of finding work for the patients, who have been received for convalescence in the Association's cottage homes, and, having no home to go to are dependent on the charity until they again become self-suporting. The present income of the Association is just over £4,000 a year and it is estimated that at least another £2,000 is needed to meet the expenses of 1923. The work has been extended to many county and borough mental hospitals with satisfactory results, and there is every prospect of greater demands being made on the Association. Subscriptions or donations therefore will be thankfully received by the secretary, Miss Vickers. Church House, Dean's Yard, Westminster, S.W.1.

**NATIONAL CHILDREN'S HOME & ORPHANAGE.**—In its 28 branches throughout the country, this institution is clothing, feeding and educating 4,000 orphan boys and girls. Special branches are set apart for the treatment of the crippled child and the child threatened with consumption. Need, not creed, decides admission. The family system is adopted throughout. the children being grouped in separate houses under the care of trained Sisters. In the various schools and workshops some 17,000 have been trained to worthy citizenship. The need of help is very urgent, and will be gladly received by the principal, Rev. W. Hudson Smith, 104-122 City Road, E.C.1.

*Note.*—The following appeal was unfortunately received too late for classification:—

**UNIVERSITY COLLEGE HOSPITAL**—The annual income of this Hospital falls short of the expenditure by some £14,000. The estimates for the year 1923 show a prospective deficit of £14,000, and. in the absence of any emergency grant or other unexpected receipt, the Hospital authorities will have seriously to consider the question of curtailing their work. Last year over 5,000 in-patients and over 66,000 out-patients were treated. Subscriptions and donations are earnestly requested to enable the Committee to carry on the work of the Hospital. Mr. J. Gerald T. Buckle, Secretary, University College Hospital, London, W.C.1.

---

### A MONTESSORI TRAINING COURS .

A training course in the principles and practice of the Montessori method of education, as applied to children from three to eleven years of age, will be conducted by Dr. Montessori in London from April 1 to end of July, 1923. Dr. Montessori will give a series of lectures on the theory of her system, and, with the help of her assistants, will give practical demonstrations of the method with groups of children. The lectures are intended primarily for teachers, but parents may also be enrolled. Recognised Montessori schools in London will be personally inspected by Dr. Montessori during the course, and either she or her assistants will conduct students on visits of observation, when questions of theory and practice will be discussed. The lectures will be delivered in Italian and repeated in English sentence by sentence. A diploma will be given to those who regularly attend, satisfactorily complete the course, and, after written and verbal examination, are deemed capable of applying the Montessori method of education to children. The tuition fee for the whole course is 35 guineas; to students holding Montessori diplomas for Infant Course, 20 guineas; to Montessori alumni, 1919 and 1921, 10 guineas. Applications for admission should be made at the earliest possible moment, on a printed form to be obtained from the hon. organiser, Mr. C. A. Bang, 20, Bedford Street, London, W.C.2.

"On the Edge of the Primeval Forest; Experiences and Observations of a Doctor in Equatorial Africa." By Prof. Albert Schweitzer, Dr. Theol, Dr. Med., Dr. Phil. (Strasbourg) Translated by Ch. Th. Campion. Illustrated. (A. & C. Black. 6s. net.) At the age of 30 Prof. Schweitzer, a well-known musician and literary man, reconstructed his life and began to study medicine. Deeply impressed by what he had read of the physical miseries o the natives of tropical Africa, he had become convinced that more medical aid should be provided by the Powers owning Colonies in that part of the world. For his own part, he had no sooner qualified than he began work on the River Ogowe in Gaboon. Here, for 4½ years, he dealt with sleeping-sickness, leprosy, elephantiasis, heart complaints, malaria, and all manner of skin diseases, helped only by his wife (a trained nurse), and a clever native, who, though he could not read, infallibly remembered the "look" of every drug label. For some time the erection of a small corrugated iron building, his only hospital was an ex-fowlhouse. To the record of his medical work Prof. Schweitzer adds much that is interesting about the country and the natives. He does not idealise the latter, nor does he advocate their Europeanisation; on the contrary, he would accept, but try to improve and refine, existing rights and customs.

